# Evaluation of semi quantitative perfusion parameter maps generated based on a fully automated non-rigid motion correction during a first pass myocardial perfusion (FPMP) MRI

**DOI:** 10.1186/1532-429X-14-S1-P253

**Published:** 2012-02-01

**Authors:** Aya Kino, Christopher Glielmi, Jeremy Collins, Mauricio S Galizia, Andrada R Popescu, Rahul Rustogi, Jacob Fluckiger, Hui Xue, Jens Guehring, Sven Zuehlsdorff, Daniel C Lee, James Carr

**Affiliations:** 1 Radiology, Northwestern University, Chicago, IL, USA; 2 Siemens Healthcare USA, Chicago, IL, USA; 3 Siemens Corporate Research USA, Princeton, NJ, USA; 4 Department of Medicine, Cardiology Division, Northwestern University/Feinberg School of Medicine, Chicago, IL, USA

## Summary

The purpose of this study is to evaluate semi quantitative perfusion parameter maps generated based on a fully automated non-rigid motion correction during a first pass myocardial perfusion (FPMP) MRI in patients with suspected coronary artery disease (CAD) or coronary micro vascular disease (CMVD).

## Background

FPMP MRI is commonly used to assess CAD and most recently to assess cardiac involvement in asymptomatic patients with CMVD such as systemic sclerosis and diabetes mellitus.

FPMP MRI evaluation relies on visual inspection for qualitative analysis but quantitative analysis of rest and stress perfusion data is desired to improve diagnosis. One main challenge of qualitative analysis includes cardiac and respiratory motion. To minimize this challenge, a previously described inline, fully automated motion correction method [Xue, H MICCAI 2009] generates a motion corrected dataset as well as pixel-wise upslope maps. Using the image at a time point selected for peak signal change during the first pass of contrast agent as the template, all other time points were registered into the template coordinate system. We compare qualitatively and quantitatively the original free breathing images and motion correted images with the corresponding maps pixel-wise upslope maps in patients with suspected CAD or CMVD.

## Methods

Seventy one patients with suspected epicardial CAD or CMVD underwent adenosine stress and rest perfusion scans on 1.5T scanner (MAGNETOM Avanto, Siemens Healthcare) using a framework to fully automatically analyze cardiac FPMP MR.

Three short axis slices were acquired during infusion of 0.075 mmol/kg of Gadolinium (Magnevist, Bayer HealthCare Pharmaceuticals, USA) adenosine (Adenoscan, Astellas Pharma, USA) infusion was administrated to induce stress. Free breathing, motion-corrected images and corresponding perfusion maps were assessed by two radiologists independently using the AHA 16 model and scored using a four point Likert scale (poor to excellent) to evaluate image quality and confidence level for the presence or absence of hypo-perfusion regions. Signal intensity curves upslope index from both free breathing and motion corrected images during stress and rest were manually calculated in non ischemic and ischemic areas and compared to the corresponding pixel wise parameter map generated based on motion corrected images.

## Results

**Figure 1:**
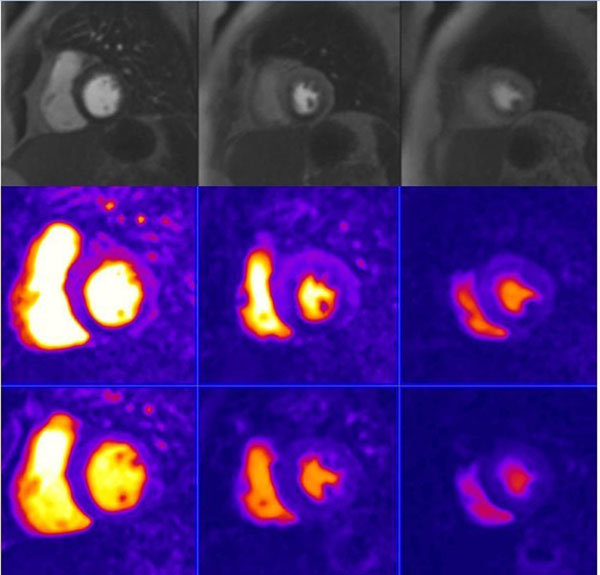
Perfusion defect is seen on stress FPMP MRI (top images)in the inferoseptum and inferior wall from base to apex and corresponding semi quantitative perfusion parameter maps (stress in the middle and rest in the botton) in a 64 years old woman with chest pain and severe stenosis in the RCA at coronary angiography.

**Figure 2:**
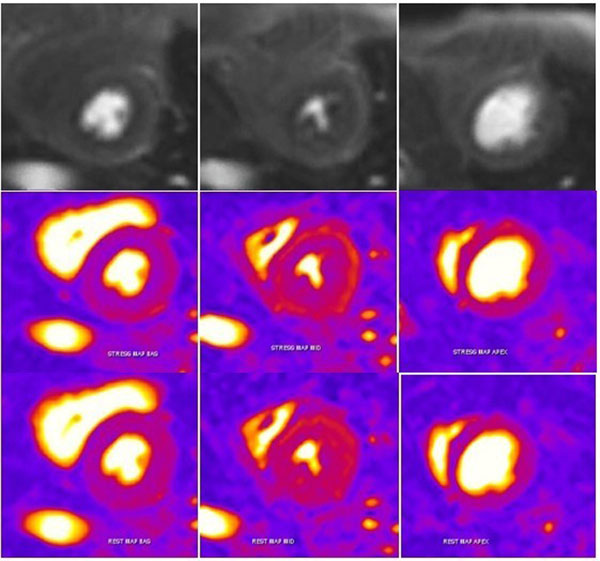
Subendocardial defect is seen on stress FPMP images (top) and corresponding semi quantitative perfusion parameter maps (stress in the middle and rest in the botton) in a 67 years old woman with history of scleroderma and normal coronary angiography.

All patients were successfully scanned. Segmental perfusion defects were identified in 27 of the 49 patients with suspected CAD patients(Fig.1) and in the remaining patients with suspected CMVD a non segmental subendocardial defect was seen in 11(Fig.2). The mean image quality score and confidence level for motion corrected images (3.58 and 3.27 respectively) were significantly higher than on free breathing images (2.96 and 3.37 respectively.Inte-reader agreement was moderate for motion corrected images and fair for free breathing images. The upslope index of non ischemic and ischemic areas and the semi quantitative perfusion parameter maps values were comparable (p<0.005).

## Conclusions

Semi quantitative perfusion parameter maps obtained by a fully automated non-rigid motion correction during a FPMP MRI correlated both qualitatively and quantitatively with the free breathing images in patients with epicardial CAD and CMVD.

## Funding

Astellas Pharma Global Development, Inc.

